# Predicting Risk of Type 2 Diabetes by Using Data on Easy-to-Measure Risk Factors

**DOI:** 10.5888/pcd14.160244

**Published:** 2017-03-09

**Authors:** Kedir N Turi, David M. Buchner, Diana S. Grigsby-Toussaint

**Affiliations:** 1 Vanderbilt University Medical Center, Nashville, Tennessee; 2 University of Illinois-Urbana Champaign, Champaign, Illinois

## Abstract

**Introduction:**

Statistical models for assessing risk of type 2 diabetes are usually additive with linear terms that use non-nationally representative data. The objective of this study was to use nationally representative data on diabetes risk factors and spline regression models to determine the ability of models with nonlinear and interaction terms to assess the risk of type 2 diabetes.

**Methods:**

We used 4 waves of data (2005–2006 to 2011–2012) on adults aged 20 or older from the National Health and Nutrition Examination Survey (n = 5,471) and multivariate adaptive regression splines (MARS) to build risk models in 2015. MARS allowed for interactions among 17 noninvasively measured risk factors for type 2 diabetes.

**Results:**

A key risk factor for type 2 diabetes was increasing age, especially for those older than 69, followed by a family history of diabetes, with diminished risk among individuals younger than 45. Above age 69, other risk factors superseded age, including systolic and diastolic blood pressure. The additive MARS model with nonlinear terms had an area under curve (AUC) receiver operating characteristic of 0.847, whereas the 2-way interaction MARS model had an AUC of 0.851, a slight improvement. Both models had an 87% accuracy in classifying diabetes status.

**Conclusion:**

Statistical models of type 2 diabetes risk should allow for nonlinear associations; incorporation of interaction terms into the MARS model improved its performance slightly. Robust statistical manipulation of risk factors commonly measured noninvasively in clinical settings might provide useful estimates of type 2 diabetes risk.

## Introduction

Consensus is lacking on whether to screen asymptomatic adults for type 2 diabetes in the United States (1,2), despite extensive work on statistical models to predict risk (3). The risk-predictive performance of these models may vary over time and between populations and geographic areas, but the models typically perform well in the populations for which they were developed (4). Eight risk models have been developed for the US population (3). Five are based on data extracted from cohort studies that largely do not reflect the racial/ethnic composition of the US population; as such, these models have limited validity. Three models, however, are based on representative US samples (5–7); each used data from the National Health and Nutrition Examination Survey (NHANES) III (1988–1994), which we also used for this study.

Diabetes prediction models usually are additive models and use linear terms (8), and most do not account for interactions between risk factors (9). If nonlinear associations and interactions between variables are ignored, the accuracy of the models may be compromised. The objectives of this study were to use US nationally representative data on diabetes risk factors and spline regression models to determine the ability of models with nonlinear terms and interaction terms to assess the risk of type 2 diabetes.

## Methods

Data were extracted from NHANES, a cross-sectional and nationally representative survey of the noninstitutionalized US civilian population. Data from 4 waves (2005–2006 to 2011–2012), excluding data for pregnant participants and those aged younger than 20 years, were combined to obtain the sample (10). The sample was composed of individuals who answered the question “Other than during pregnancy, have you ever been told by a doctor or health professional that you have diabetes or sugar diabetes?” or those who had valid laboratory test results for diabetes and consisted of 22,660 observations. We then excluded participants who were missing data on 1 or more of the 17 independent variables examined; the final sample consisted of 5,471 participants.

### Dependent variable

The classification of survey participants into 2 groups — those who had type 2 diabetes and those who did not — was based on survey questions and biochemical measures. Survey participants were considered to have diabetes if they 1) answered yes to the question “Other than during pregnancy, have you ever been told by a doctor or health professional that you have diabetes or sugar diabetes?”; 2) answered no to the preceding question but had a measured fasting plasma glucose of 126 mg/dL or greater or a measured HbA1c of 6.5% or more (ie, undiagnosed diabetes); or 3) self-reported diagnosed diabetes but had a measured fasting plasma glucose of less than 126 mg/dL and a measured HbA1c of less than 6.5% (which did not conform with the self-reported results) and reported taking insulin or oral hypoglycemic medication. This method for classifying diabetes status was used in previous studies (11,12) and is the most accurate method of ascertaining type 2 diabetes; it has a sensitivity of approximately 95% in NHANES data (13). Participants who did not self-report a diagnosis of diabetes, who had a measured fasting plasma glucose of less than 126 mg/dL and a measured HbA1c of less than 6.5%, and who reported not taking medications were classified as not having type 2 diabetes. Building on previous work (14), we classified survey participants as not having type 2 diabetes if they responded no to the self-reported diabetes diagnosis question but self-reported taking insulin or oral hypoglycemic medications.

Although type 1 diabetes and type 2 diabetes are distinct diseases, NHANES data do not clearly differentiate between them. We followed a method outlined previously (12) to differentiate between type 1 diabetes and type 2 diabetes in those who self-reported a diagnosis of diabetes. Accordingly, 15 participants who reported receiving a diagnosis of diabetes when they were younger than 30 years and were using insulin only to manage their diabetes were classified as having type 1 diabetes and excluded from further analysis. Participants who reported receiving a diagnosis of diabetes when they were younger than 30 years and were using both insulin and hypoglycemic medication or only hypoglycemic medication to manage their diabetes were classified as type 2 diabetes. All participants who self-reported receiving a diagnosis of diabetes when they were 30 years or older were classified as having type 2 diabetes.

### Independent variables

The following demographic variables were treated as covariates: age, sex, marital status, and race/ethnicity (Table 1). The age range was 20 to 84 years (mean, 46.7 y); 45.9% of the sample were men. Self-reported race/ethnicity was categorized as Mexican American (17.9%), other Hispanic (10.5%), black (18.2%), non-Hispanic white (48.8%), and others (including Asians, American Indians, and multiracial, 4.6%). Marital status was categorized as never married, married or living with a partner, and separated, divorced or widowed.

**Table 1:** Distribution of Variables for the Study Sample^a^ (N = 5,471), National Health and Nutrition Examination Survey (NHANES), 2005–2012

Characteristic	Total No. (%^a^)	Has Diabetes	
		No (%^a^)	Yes (%^a^)
**Sex**			
Male	2,510 (45.9)	2,182 (46.0)	328 (45.1)
Female	2,961 (54.1)	2,561(54.0)	400 (54.9)
All	5,471 (100.0)	4,743 (100.0)	728 (100.0)
**Race/ethnicity**			
Mexican American	984 (17.9)	818 (17.3)	166 (22.8)
Other Hispanic ethnicity	572 (10.5)	486 (10.3)	86 (11.8)
Non-Hispanic white	2,668 (48.8)	2,360 (49.8)	308 (42.3)
Black	994 (18.2)	849 (17.9)	145 (19.9)
Others (Asian, American Indian, multiracial)	253 (4.6)	230 (4.9)	23 (3.2)
All	5,471 (100.0)	4,743 (100.0)	728 (100.0)
**Marital status**			
Never married	1,076 (19.7)	1,009 (21.3)	67 (9.2)
Married or living with partner	3,181 (58.1)	2,778 (58.6)	403 (55.4)
Separated, divorced, or widowed	1,214 (22.2)	956 (20.1)	258 (35.4)
All	5,471 (100.0)	4,743 (100.0)	728 (100.0)
**Household food security^b^**			
Full food security	3,818 (69.8)	3,297 (69.5)	521 (71.6)
High marginal food security	592 (10.8)	521 (11.0)	71 (9.8)
Low food security	675 (12.3)	596 (12.6)	79 (10.8)
Very low food security	386 (7.1)	329 (6.9)	57 (7.8)
All	5,471 (100.0)	4,743 (100.0)	728 (100.0)
**Education level**			
Less than high school diploma	1,523 (27.8)	1,218 (25.7)	305 (41.9)
High school diploma	1,326 (24.2)	1,139 (24.0)	187 (25.7)
Some college or higher	2,622 (47.9)	2,386 (50.3)	236 (32.4)
All	5,471 (100.0)	4,743 (100.0)	728 (100.0)
**Cigarette smoking^c^**			
Never smoker	3,804 (69.5)	3,272 (69.0)	532 (73.1)
Former smoker	255 (4.7)	231 (4.9)	24 (3.3)
Current smoker	1,412 (25.8)	1,240 (26.1)	172 (23.6)
All	5,471 (100.0)	4,743 (100.0)	728 (100.0)
**Alcohol consumption^d^**			
Abstainers	841 (15.4)	682 (14.4)	159 (21.8)
Occasional drinker	2,545 (46.5)	2,192 (46.2)	353 (48.5)
Moderate drinker	901 (16.5)	834 (17.6)	67 (9.2)
Heavy drinker	1,184 (21.6)	1,035 (21.8)	149 (20.5)
All	5,471 (100.0)	4,743 (100.0)	728 (100.0)
**Do you have close relative who has/had diabetes?**			
No	3,321 (60.7)	3,039 (64.1)	282 (38.7)
Yes	2,150 (39.3)	1,704 (35.9)	446 (61.3)
All	5,471 (100.0)	4,743 (100.0)	728 (100.0)
**Continuous variables, mean (SD)**			
Age, y	46.7 (17.4)	44.6 (16.9)	61.1 (13.1)
Body mass index, kg/m^2^	27.5 (5.6)	27.1 (5.4)	30.0 (5.8)
Waist circumference, cm	95.0 (13.9)	93.7 (13.6)	103.4 (13.2)
Subscapular skinfold, mm	21.3 (8.2)	20.8 (8.2)	24.5 (7.7)
Family income-to-poverty ratio^e^	2.5 (1.6)	2.5 (1.6)	2.2 (1.5)
Depression, mean score^f^	2.6 (3.7)	2.5 (3.6)	3.1 (4.3)
Moderate-to-vigorous physical activity, no. of min/d^g^	101.7 (157.1)	108.0 (160.9)	60.6 (121.9)
Diastolic blood pressure, mm Hg	69.6 (11.7)	69.7 (11.5)	68.9 (13.0)
Systolic blood pressure, mm Hg	121.8 (18.3)	120.3 (17.4)	131.6 (20.8)
Sleep duration, h	6.8 (1.4)	6.8 (1.4)	6.7 (1.6)

Abbreviations: SD, standard deviation.

a Data from 4 waves (2005–2006 to 2011–2012), excluding data for pregnant participants and those aged younger than 20 years, were combined to obtain the sample. The sample was composed of those who answered the question “Other than during pregnancy, have you ever been told by a doctor or health professional that you have diabetes or sugar diabetes?” or those who had valid laboratory test results for diabetes; the sample consisted of 22,660 observations. We then excluded participants who were missing data on 1 or more of the 17 independent variables examined; the final sample consisted of 5,471 participants.

b NHANES assesses household food security through a 10-item questionnaire and classifies adults into 4 categories: 1) full food security (no food security concerns), 2) marginal food security (1 or 2 concerns), 3) low food security (3–5 concerns), and very low food security (6–10 concerns).

c Participants who self-reported smoking at least 100 cigarettes in their lifetime and smoking every day or some days at the time of the interview were classified as current smokers. Participants who reported smoking at least 100 cigarettes in their lifetime and not smoking at all at the time of the interview were classified as ex-smokers. Participants who reported smoking fewer than 100 cigarettes in their lifetime were classified as never-smokers.

d Categorized as abstainer (<12 alcoholic drinks in lifetime; 15.4%), occasional drinker (≤1 alcoholic drink per week in previous 12 months, 46.5%), moderate drinker (2 or 3 alcoholic drinks per week in previous 12 months; 16.5%), and heavy drinker (>3 alcoholic drinks per week in previous 12 months, 21.6%) (16).

e Calculated by dividing family income by the federal poverty threshold specific to household size and year. A ratio of 1.0 or greater indicates income above the poverty level.

f Computed from the Patient Health Questionnaire-9 (19); scored from 0 to 36, with higher scores indicating higher levels of depression.

g An estimate of average daily time spent in moderate-to-vigorous physical activity (MVPA) was computed by adding up the minutes of reported MVPA during transportation, work, and leisure activities from 7 days of recall and dividing the total number of minutes by 7.

We measured overall adiposity, visceral adiposity, and back-of-the-trunk subcutaneous adiposity: body mass index (BMI, kg/m^2^), ranged from 13.0 to 57.0 (mean, 27.5); waist circumference, ranged from 59.7 cm to 166.4 cm (mean, 95.0 cm); and subscapular skinfold, ranged from 4.4 mm to 42.0 mm (mean, 21.3 mm).

An estimate of average daily time spent in moderate-to-vigorous physical activity (MVPA) was computed by adding up the minutes of reported MVPA during transportation, work, and leisure activities from 7 days of recall and dividing the total number of minutes by 7. MVPA ranged from 0 to 1,371 minutes per day (mean, 101.7 min/d).

Participants who self-reported smoking at least 100 cigarettes in their lifetime and smoking every day or some days at the time of the interview were classified as current smokers. Participants who reported smoking at least 100 cigarettes in their lifetime and not smoking at all at the time of the interview were classified as ex-smokers. Participants who reported smoking fewer than 100 cigarettes in their lifetime were classified as never-smokers. This smoking classification is widely used (15). Accordingly, 69.5% of the sample were never-smokers, 4.7% were ex-smokers, and 25.8% were current smokers. The consumption of alcohol was categorized into abstainers (<12 alcoholic drinks in lifetime; 15.4%), occasional drinkers (≤1 alcoholic drink per week in previous 12 months, 46.5%), moderate drinkers (2 or 3 alcoholic drinks per week in previous 12 months; 16.5%), and heavy drinkers (>3 alcoholic drinks per week in previous 12 months, 21.6%) (16). Self-reported family history of diabetes was categorized as a yes or no dummy variable. Approximately 39.3% of the sample indicated having at least 1 relative with diabetes.

The depression score was computed from the Patient Health Questionnaire-9 (PHQ-9 [17]). The score, which helps to define the severity of depression relevant as a risk factor for type 2 diabetes, was used as a continuous variable. Each of the 9 items, which assess the frequency a person experiences 9 symptoms of depression, is scored as 0 (not at all) to 3 (nearly every day), for a possible score ranging from 0 to 36. The depression score for this sample ranged from 0 to 24 (mean 2.6). The NHANES question on sleep duration asked, “How much sleep do you usually get at night on weekdays or workdays?” The mean sleep duration in our sample was 6.8 hours per day. Systolic blood pressure ranged from 78 mm Hg to 228 mm Hg (mean, 121.8 mm Hg), and diastolic blood pressure ranged from 10 mm Hg to 132 mm Hg (mean, 69.6 mm Hg).

The socioeconomic covariates were education level and family income-to-poverty-ratio; the latter was calculated by dividing family income by the federal poverty threshold specific to household size and year. A ratio of 1.0 or greater indicates income above the poverty level. The mean income-to-poverty-ratio was 2.5 and ranged from 0 to 5.0. NHANES assesses household food security through a 10-item questionnaire and classifies adults into 4 categories: 1) full food security (no food security concerns), 2) marginal food security (1 or 2 concerns), 3) low food security (3–5 concerns), and very low food security (6–10 concerns). Accordingly, 69.8% of the study participants had full food security, 10.8% marginal food security, 12.3% had low food security, and 7.1% had very low food security. Education level was categorized as less than a high school diploma (27.8%), high school diploma (24.2%), and some college or higher (47.9%).

### Statistical analysis

Multivariate adaptive regression splines (MARS) was used to build a diabetes risk-prediction model. MARS, a type of algorithmic modeling, was selected because it accounts for both linear and nonlinear relationships as well as piecewise interactions between predicting variables. It is a powerful method that is rarely used in public health. This approach enabled us to model the interaction effect of predicting variables because it segments variables into pieces known as *basis* *functions*, or *splines*. MARS is better than typical regression models because it can handle studies with many predictors and can account for nonlinearity, multicollinearity, and a high degree of interaction among predictors (18).

Observations were randomly split into model-building data (75%) and test data (25%). The MARS model was trained and validated using a 5-fold cross-validation technique on model-building data (75% of the data). The prediction error was averaged across all out-of-fold predictions. The out-of-fold *R* ^2^ was averaged (cross-validated *R* ^2^) from the left-out subset, which is an estimate of model. performance on independent data (18). The generalized *R* ^2^ was based on the generalization cross-validation criterion incorporated into the model to avoid overfitting and directly reflects model performance.

Finally, the validated model was tested on test data (25% of the data), and the performance of the model was evaluated by measuring area under curve (AUC) receiver operating characteristic (19). The larger the AUC, the better the model performance; AUC values range from 0.5 (no better accuracy than chance) to 1.0 (perfect accuracy). The test developed by DeLong et al (20) was used to evaluate the statistical difference between the models. In addition, the improvement in classification was evaluated by using 1) net reclassification index, which quantifies how well a new model, compared with a previous model, reclassifies participants, and 2) integrated discrimination improvement, which quantifies how well a new model (with added variables), compared with a previous model (with fewer variables), predicts a binary outcome of interest (21). 

Model building began with 17 variables that are associated with an increased probability of diabetes but do not involve invasive measurement procedures. The initial model included age, sex, race/ethnicity, marital status, education level, family history of diabetes, cigarette smoking, alcohol consumption, household food security, depression score, family income-to-poverty ratio, waist circumference, BMI, subscapular skinfold, systolic and diastolic blood pressure, sleep duration, and MVPA. The baseline model for the performance of MARS models was a logistic regression model.

The additive MARS model showed that the forward selection process transformed the variables into 35 basis functions with 13 variables, including the intercept. The variables in the forward selection process were age, waist circumference, family history of diabetes, race/ethnicity, family income-to-poverty ratio, education level, BMI, subscapular skinfold, systolic blood pressure, diastolic blood pressure, MVPA, sleep duration, and depression score. The forward MARS selection process overfitted the prediction model; thus, in a backward selection process, we removed 1 basis function at a time. The final additive MARS model had 21 basis functions but still comprised the same 13 variables in the forward selection process. 

The second model incorporated 2-way interactions between basis functions. The final selection process produced 28 basis functions, including the constant. The interactions between the 12 pairs of basis functions were incorporated into the final model, which had 20 basis functions. Eight variable basis interactions included in the model are: waist circumference and subscapular skin fold, age and systolic blood pressure, depression score and diastolic blood pressure, race/ethnicity and diastolic blood pressure, race/ethnicity and sleep duration, education and alcohol consumption, race/ethnicity and age, and BMI and systolic blood pressure.

## Results

### Additive MARS model

In the additive MARS model, we observed a high risk of diabetes at ages older than 69 (Table 2). However, the importance of age as a predictor declined after age 45, and other factors became more important. The data for systolic blood pressure reflected a *J*-shaped curve: the risk of diabetes slightly decreased below 125 mm Hg, did not increase between 125 mm Hg to 158 mm Hg, and then started to spike at 158 mm Hg. On the other hand, diastolic blood pressure reflected a* U*-shaped curve: the risk of diabetes increased below 55 mm Hg and above 83 mm Hg. For MVPA, the risk of diabetes decreased starting at 29 minutes of MVPA per day. For sleep duration, the risk of diabetes increased at less than 7 hours. A depression score of 11 or more increased the risk of diabetes.

**Table 2:** Results of Additive MARS Model^a^ After Backward Selection and 2-Way Interaction MARS Model^b^, National Health and Nutrition Examination Survey (N = 5,471), 2005–2012^c^

Variable/Basis Function	Coefficient
**Additive MARS Model^a^**	
**Intercept**	4.40
**Age**	
Max (age [y] − 69, 0)	0.07
Max (69 − age [y], 0)	−0.11
Max (age [y] − 45, 0)	−0.06
**No family history of diabetes**	−1.03
**White race**	−0.51
**Education is less than high school diploma**	0.34
**Adiposity**	
Max (body mass index − 48.2, 0), kg/m^2^	0.32
Max (48.2 − body mass index, 0), kg/m^2^	0.06
Max (141.4 − waist circumference, 0), cm	−0.05
Max (27.7 − subscapular skinfold, 0), cm	−0.06
**Depression^d^**	
Max (depression score − 13, 0)	−0.82
Max (depression score − 11, 0)	0.63
**Blood pressure, mm Hg**	
Max (diastolic blood pressure − 83.3, 0)	0.19
Max (83.3 − diastolic blood pressure, 0)	−0.05
Max (diastolic blood pressure − 91.3, 0)	−0.13
Max (diastolic blood pressure − 55.3, 0)	−0.09
Max (124.6 − systolic blood pressure, 0)	−0.02
Max (systolic blood pressure − 158, 0)	0.02
**Moderate–to-vigorous physical activity, min/d**	
Max (28.6 − moderate-to-vigorous physical activity, 0)	0.01
**Sleep duration, h**	
Max (7 − sleep duration, 0)	0.11
**2-Way Interaction MARS Model^b^**	
**Intercept**	1.85
Max (age − 69, 0), y	−0.22
Max (69 − age, 0), y	−0.03
No family history of diabetes	−0.56
Max (141.3 − waist circumference, 0), cm	−0.03
Max (subscapular skinfold − 11.1, 0), mm	0.03
Max (11.1 − subscapular skinfold, 0), mm	−0.19
Max (body mass index − 21.9, 0), kg/m^2^	−0.13
Max (21.9 − body mass index, 0), kg/m^2^	−0.51
Max (family income-to-poverty ratio^e^ − 0.77, 0)	−0.19
Max (0.77 − family income-to-poverty ratio^e^, 0)	−1.29
Max (age − 69, 0), y × max (waist circumference − 123.4, 0), cm	0.19
Max (age − 69, 0), y × max (123.4 − waist circumference, 0), cm	0.005
Max (69 − age, 0), y × max (systolic blood pressure − 190, 0), mm Hg	0.005
Max (69 − age, 0), y × max (190 − systolic blood pressure, 0), mm Hg	−0.0007
Max (age − 69, 0), y × max (body mass index − 18.9, 0), kg/m^2^	0.014
Max (age − 69, 0), y × max (18.9 − body mass index, 0), kg/m^2^	0.20
Max (69 − age, 0), y × max (body mass index − 24.5, 0), kg/m^2^	0.007
Max (69 − age, 0), y × max (24.5 − body mass index, 0), kg/m^2^	0.009
Max (21.9 − body mass index, 0), kg/m^2^ × max (53 − age, 0), y	−0.008
Max (21.9 − body mass index, 0), kg/m^2^ × max (family income-to-poverty ratio^e^ − 0.3, 0)	−0.19
Max (21.9 − body mass index, 0), kg/m^2^ × max (0.3 − family income-to-poverty ratio^e^, 0)	3.007
Max (family income-to-poverty ratio^e^ − 0.77, 0) × max (diastolic blood pressure − 79.3, 0), mm Hg	0.02
Max (family income-to-poverty ratio^e^ − 0.77, 0) × max (79.3 − diastolic blood pressure, 0), mm Hg	0.007
Max (141.3 − waist circumference, 0), cm × max (12 − depression score^d^, 0)	−0.001
Has a family history of diabetes × Mexican American ethnicity	0.64
Has a family history of diabetes × max (79.3 − diastolic blood pressure, 0), mm Hg	0.02
Has a high school diploma × max (141.3 − waist circumference, 0), cm	−0.01

Abbreviations: MARS, multivariate adaptive regression splines; max, maximum.

a Additive MARS does not incorporate interactions between basis functions.

b 2-way MARS model features interactions between 2 basis functions. A spline is a smooth piecewise polynomial (an expression of >2 algebraic terms); knots are the places where the polynomials join.

c Example of an interpretation of basis function: at each knot of a variable (for example, 69 y and 45 y for age) there are 2 basis functions that reflect each other. Both basis functions could be significant, or only one could be significant. Age <69 y and age >69 y are both significant and have linear coefficients of −0.11 and 0.07, respectively. In the same variable at a knot value of 45, only 1 basis function is significant (age >45 y) and is included in the model; it has a linear coefficient of −0.06. If age does not fall into these ranges (for example, if age <69 for basis function max [age, y − 69, 0]), it would be assigned a value of 0; thus max (age, y − 69, 0) indicates this mathematical relationship.

d Computed from the Patient Health Questionnaire-9 (17); scored from 0 to 36, with higher scores indicating higher levels of depression.

e Calculated by dividing family income by the federal poverty threshold specific to household size and year. A ratio of 1.0 or greater indicates income above the poverty level.

### Two-way interaction MARS model

**Figure 1:**
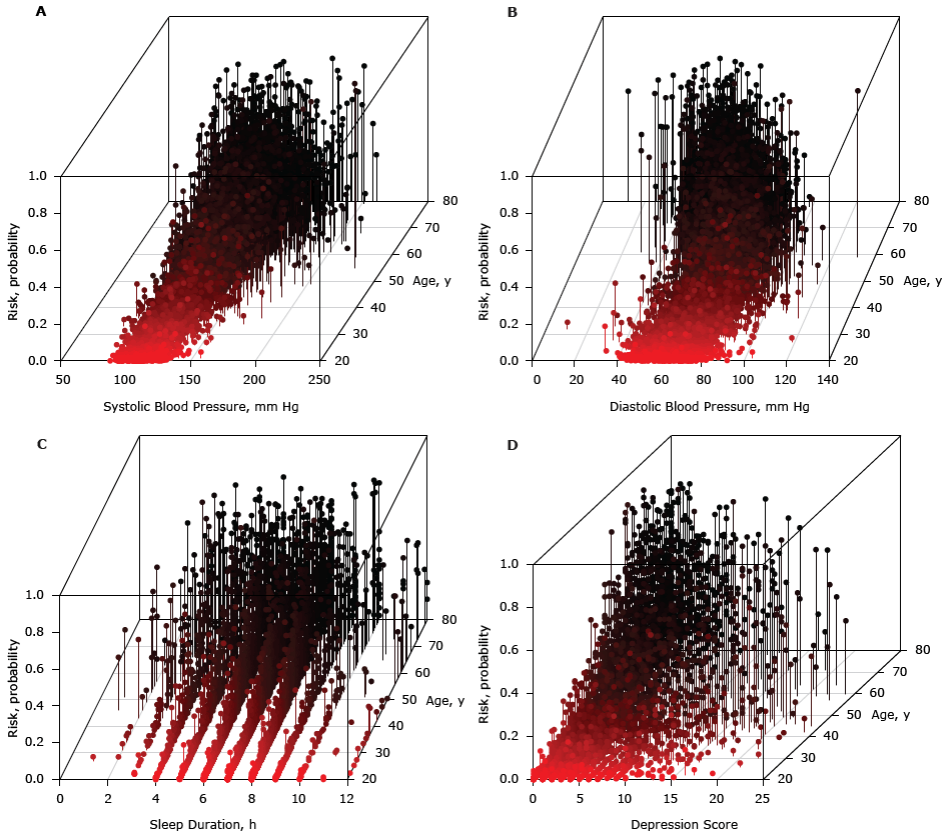
A) Contribution of interaction between systolic blood pressure and age to the risk of diabetes, B) Contribution of interaction between diastolic blood pressure and age to the risk of diabetes, C) Contribution of interaction between age and sleep duration to the risk of diabetes, and D) Contribution of interaction between age and depression score to the risk of diabetes.

The interaction between a waist circumference greater than 123.4 cm and an age greater than 69 increased the risk of diabetes (Table 2). The interaction between an age younger than 69 and a systolic blood pressure greater than 190 mm Hg increased the risk of diabetes (Figure 1A). The greatest risk of diabetes was at the intersection of a diastolic blood pressure of 50 mm Hg and age older than 60 (Figure 1B). The highest risk was at an age older than 69 and a systolic blood pressure above 200 mm Hg. The interaction between a lower range of diastolic blood pressure (<69 mm Hg) and older age (>50 y) increased the risk of diabetes. The interaction between a depression score of less than 12 and a waist circumference of less than 141 cm decreased the risk of diabetes. The following interactions decreased risk: having a family history of diabetes and being Mexican American, having a family history of diabetes and a diastolic blood pressure less than 79 mm Hg, and having at least a high school diploma and a waist circumference of less than 141 cm. Additionally, even though the interaction basis functions were not significant in the final 2-way MARS model, both short and long hours of sleep (Figure 1C) and high depression score (Figure 1D) at older age increased diabetes risk. 

### Model performance

**Figure 2:**
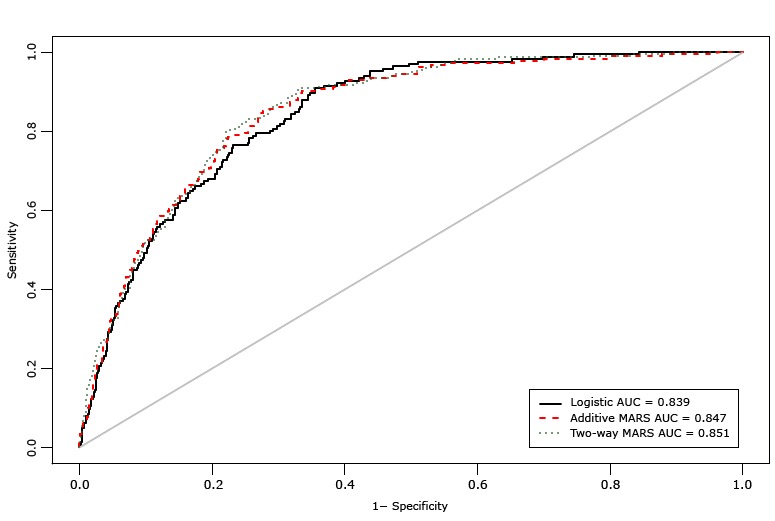
Area under the receiver operating characteristics curve (AUC) comparing 3 diabetes risk-prediction models: a logistic regression model, an additive MARS model, and 2-way interaction MARS model. Abbreviation: MARS, multivariate adaptive regression splines.

The AUC for the additive MARS model was 0.847 (95% confidence interval, 0.819–0.875) and for the 2-way interaction MARS model was 0.851 (95% confidence interval 0.827–0.876) (Figure 2). The difference between the AUC for the additive MARS model and the AUC for the logistic regression model was not significant, whereas the difference between the AUC for the 2-way interaction MARS model and the AUC for the additive MARS model was significant (χ^2^ = 5.19, *P* = .02). The additive MARS model and 2-way interaction MARS model each had 87% accuracy. In addition, net reclassification index and integrated discrimination improvement matrices also showed significant improvement of the MARS models over the logistic model in the classification of diabetes risk status (Table 3).

**Table 3:** Comparison of Logistic Regression Model, Additive MARS Model, and 2-Way Interaction MARS Model Using Net Reclassification Index^a^ and Integrated Discrimination Improvement^b^

Criteria	Logistic vs Additive^c^ MARS	Logistic vs 2-Way MARS^c^	Additive MARS^c^ vs 2-Way MARS^d^
NRI (95% CI) [*P* value]	0.5 (0.2 to 0.8) [.004]	0.03 (−0.3 to 0.3) [.80]	−0.2 (−0.5 to 0.08) [.15]
IDI (95% CI) [*P* value]	0.05 (0.006 to 0.08) [.02]	−0.04 (−0.06 to −0.008) [.01]	0.04 (−0.08 to 0.004) [.08]

Abbreviations: CI, confidence interval; IDI, integrated discrimination improvement; MARS, multivariate adaptive regression splines; NRI, net reclassification index.

a NRI quantifies how well a new model, compared with a previous model, reclassifies participants.

b IDI quantifies how well a new model (with added variables), compared with a previous model (with fewer variables), predicts a binary outcome of interest.

c Additive MARS does not incorporate interactions between basis functions.

d 2-Way MARS model features interactions between 2 basis functions.

## Discussion

The accuracy and AUC of the MARS models were higher than those for the logistic regression model and for models described in similar studies using noninvasive measurements, such as the model described by Kahn et al (22), which had an AUC of 0.71; the model described by Schmidt et al (23), which had an AUC of 0.71; and the model described by Bang et al (5), which had an AUC of 0.79. The additive MARS model had the same AUC of 0.85 as the model described by Heikes et al (7); this latter study implemented a classification and regression tree method on NHANES III data. The predictive capacity of the additive MARS model was better than or similar to the predictive capacity of models that used data obtained through invasive measures, such as taking blood samples to determine lipid profiles. Such invasive models have AUCs ranging from 0.73 to 0.85 (6,24–26).

In the 2-way interaction MARS model, 2-way interactions between the basis functions showed a slight improvement over the additive MARS model. The difference in the AUC between the MARS models and the logistic regression model was significant. The performance level of the MARS models is a new achievement in invasive and noninvasive diabetes risk-prediction models in the United States.

More importantly, the MARS models identified nonlinear and interactive relationships between some of the predicting variables and the risk of diabetes. Among the most important nonlinear associations were the relationships of systolic blood pressure and diastolic blood pressure to the risk of diabetes, which are *J*-shaped and *U*-shaped, respectively. However, the cutoff point for blood pressure control in people with diabetes and people without diabetes is being debated. The United Kingdom Prospective Diabetes Study showed that patients with tight blood pressure control (a mean of 144/82 mm Hg) reduced the number of macrovascular and microvascular events (27). Recent meta-analyses and a Cochrane systematic review of randomized control studies and cohort-based observational studies did not find evidence for tight blood pressure control of less than 130/80 mm Hg, as recommended in some guidelines (28,29).

The *U*-shaped association between sleep and diabetes risk observed in our study, where sleep duration of less than 7 hours or more than 8 hours increases the risk of diabetes, is similar to the association found by Yaggi et al (30). However, our study found that less than 7 hours was a natural cutoff point for short sleep, whereas Yaggi et al found 6 hours to be a cutoff (30); thus, shorter sleep is more predictive than longer sleep of diabetes risk. Moreover, the effect of sleep duration is mediated by race/ethnicity and age, and greater waist circumference, higher systolic blood pressure, and lower diastolic blood pressure are mediated by age. In addition, having a family history of diabetes makes it more difficult for an individual to reduce the risk of diabetes through physical activity.

The strengths of this study are that the model used noninvasive measurements. The information used for this model can be obtained through surveys and nonclinical measurements in nonclinical settings. Another strength is that the NHANES data used are representative of the US population. In addition, the MARS models add new, never-used-before information as diabetes prediction markers, such as depression score and sleep duration. The performance of the model decreased when these factors were excluded. This study also has limitations. First, the prediction model was not validated on an independent and representative sample outside NHANES. Second, our model was built on the complete case analysis; data may not be missing completely at random. In addition, NHANES data are research data; trained personnel accurately and repeatedly collect the anthropometric and blood pressure measurements. At the community level, such trained personnel and other resources may not be available, so replicating research standards of accuracy at the community level may be challenging. Finally, the study could be replicated as a more purely predictive model by using data on respondents who were classified as having type 2 diabetes per laboratory testing and who had not previously received a diagnosis.

The goal of our study was to improve the performance of diabetes prediction models. Our results suggest that statistical models of type 2 diabetes risk should allow for nonlinear associations and that the incorporation of interaction terms in the models may not improve their predictive ability. The results confirm previous findings that useful estimates of the risk of type 2 diabetes can be derived from risk factors commonly measured noninvasively in clinical settings. The MARS method produced additive and interaction models with a predictive ability that exceeds that of previously described prediction models. The nonlinearity of the model helped identify cutoff points for some risk factors, such as systolic blood pressure and diastolic blood pressure, sleep duration, and waist circumference. In short, the model maximized prediction from measurements that were noninvasive and feasible to measure in nonclinical settings. However, the practical application of this model as a screening tool needs to be replicated and further developed, particularly in community settings.
